# Editorial: Urology Mini Symposium

**Published:** 1992-12

**Authors:** Julian Kabala


### Editorial : Urology Mini Symposium

In common with many other specialities in medicine urology continues not only to evolve but to do so at an increasingly rapid pace. Diseases of the urinary tract are an extremely common cause of morbidity and in some respects are likely to become more so. With the rising proportion of the population represented by the elderly, manifestations of urological conditions in this age group is already a matter of considerable importance and an example of an intrinsically important area is elegantly discussed in Dr Tobin's article on incontinence in the elderly.

Overlapping with the theme of morbidity in the elderly but extending into the main stream of urology is the numerically huge problem of benign prostatic hyperplasia. Current strands in the pathophysiology and treatment of this complex condition is discussed in depth in Mr McLoughlan's article on this subject.

The remaining three articles (on cancer of the prostate, cancer of the bladder and ultrasound in scrotal disorders) have a strong oncological bias. They address three important trends that can be seen in other fields as well as urology. Firstly modern imaging techniques, particularly the cross-sectional imaging modalities of ultrasound, computed tomography and (still in its relative infancy) magnetic resonance imaging have had an enormous impact on the staging and follow up of tumours and will no doubt occupy an increasingly important position in the future. Secondly the newer reconstructive and conservative surgical techniques offer technical challenges to the surgeon, potentially great benefits to the patient and place great emphasis on accurate imaging both preoperatively and for purposes of follow up. Finally early detection of tumours, particularly carcinoma of the prostate is an issue that has featured heavily in the literature recently combining many of the themes discussed above as well as representing a potentially daunting demand on available resources.

It is against the background of these trends that I hope the articles that follow will offer a glimpse into these exciting areas in an evolving and modern speciality.

Julian Kabala

